# Cytokine profile, proliferation and phosphorylation of ERK1/2 and Akt in circulating mononuclear cells from individuals during the chronic intestinal phase of *Schistosomiasis mansoni* infection

**DOI:** 10.1186/1471-2334-12-380

**Published:** 2012-12-27

**Authors:** Roberta Oliveira-Prado, Iramaya Rodrigues Caldas, Andréa Teixeira-Carvalho, Marcus Vinicius Andrade, Rafaelle Christine Gomes Fares, Laís Maroni Portugal, Andréa Gazzinelli, Rodrigo Corrêa-Oliveira, José Renan Cunha-Melo

**Affiliations:** 1 Centro de Pesquisas René Rachou, Fundação Oswaldo Cruz, Belo Horizonte, MG, Brazil; 2 Fundação Oswaldo Cruz, Brasilia, DF, Brazil; 3 Departamento de Clínica Médica, Cirurgia, Faculdade de Medicina, Universidade Federal de Minas Gerais (UFMG), Belo Horizonte, MG, 30130-100, Brazil; 4 Escola de Enfermagem, Universidade Federal de Minas Gerais (UFMG), Belo Horizonte, MG, Brazil; 5 Departamento de Cirurgia, Faculdade de Medicina, Universidade Federal de Minas Gerais (UFMG), Av. Alfredo Balena, 190, sala 295, 30130-100, Belo Horizonte, MG, Brazil; 6 Instituto Nacional de Ciência e Tecnologia em Doenças Tropicais - INCT-DT, Belo Horizonte, Brazil

**Keywords:** Schistosomiasis mansoni, PBMC, Th1 and Th2 cytokines, ERK1/2, Akt

## Abstract

**Background:**

The immune response to *Schistosoma mansoni* is characterized by a granulomatous reaction around the parasite eggs that are trapped in the host liver, and this reaction modulates the immune response during the chronic phase of the disease. The typical peripheral blood mononuclear cell (PBMC) response of patients during the chronic intestinal phase of infection is characterized by a decreased response to an *S. mansoni* soluble egg antigen. To obtain a greater understanding of *Schistosoma* infections, this study investigated the effects of the soluble egg antigen (SEA) and soluble adult worm antigen (SWAP) of *S. mansoni* on cellular proliferation, cytokine production, and ERK1/2 and Akt phosphorylation in PBMCs from infected (XTO) and egg-negative (NI) individuals living in the same endemic area.

**Methods:**

The activation status was evaluated by cell immunophenotypic staining (cytometry). The cell proliferation assay was by CFSE method. Cytokine detection assay (Th1 and Th2) was by Cytometric Bead and Array phosphorylation status was by ELISA.

**Results:**

The XTO, NI and BD (blood donor) individuals from an area not endemic for schistosomiasis were compared. The CD4^+^ T lymphocyte proliferation rate was lower in the XTO group, but not the NI group, after SEA stimulation compared to the BD group. The CD8^+^ T cell proliferation rate was lower in the XTO group in the unstimulated cultures and after both SEA and SWAP stimulation compared to the BD group. Cytokine analysis after either SEA or SWAP stimulation showed a balanced cytokine pattern in the XTO and NI groups. ERK1/2 and Akt phosphorylation were only marginally detected in all groups; however, a decrease in ERK 1/2 phosphorylation was observed in the SWAP-stimulated XTO group compared to both the NI and BD groups.

**Conclusions:**

The data indicate that SEA-stimulated CD4^+^ T cells from infected patients have a lower proliferation rate than the same cells from the NI group. Furthermore, we observed that SWAP stimulation influences ERK1/2 phosphorylation in the XTO group.

## Background

Granuloma modulation by the eggs of *Schistosoma mansoni* is observed during the transition from the acute phase to the chronic phase in infected individuals*.* The exudative-necrotic granuloma of the acute phase becomes smaller and contains fewer inflammatory cells. This lack of inflammatory cells appears to be less pathogenic to the liver cells [1-5]. *Schistosoma* infection causes a range of morbidities, which is influenced to a large extent by the nature of the induced immune response and its effects on granuloma formation. By contrast, field studies in endemic areas and experimental data have led to the hypothesis that the immune response is influenced by host genetics, parasite burden, *in utero* sensitization to *Schistosoma* antigens and co-infection status [6].

The relationship between the development of the immune response and disease severity has been studied. During the chronic phase of *S. mansoni* infection, the worms and their antigens interact with the host immune response by down-regulating T-cell responses [2,7-9]. Extensive studies have examined the immunomodulation of peripheral blood mononuclear responses to *Schistosoma* antigens in infected patients. A typical PBMC response in patients during the chronic intestinal stage is characterized by lower anti-SEA (soluble egg antigen) responsiveness in contrast to higher anti-SWAP (soluble worm antigen preparation) responsiveness [2,3].

A number of cellular mechanisms have been proposed to explain the down-regulation that occurs in chronic infections. Among these hypotheses, we are focused on the role of cytokines [10,11] and distinct T-cell subsets and their activation states [12,13]. A mixed Th1-Th2 response, with a predominance of Th2 cytokines, is generally observed in chronically infected patients [14-16].

The intracellular pathways involved in PBMC responses to *S. mansoni* antigens are not well known. One of the first phenomena to occur after receptor activation is the phosphorylation of the tyrosine residues of numerous intrace-llular proteins. The earliest event is activation of the Src family protein tyrosine kinases, Lck and Fyn, which subsequently phosphorylate the immunoreceptor tyrosine-based activation motifs (ITAMs) present in the ζ and CD3 ε, δ, and γ subunits of the TCR. Phosphorylated ITAMs promote recruitment and subsequent activation of ZAP-70 [17]. The activation of ZAP-70 is implicated in the T cell receptor signal transduction pathway and IL-2 production [18].

The activation of mitogen-activated protein kinases (MAPK) is also part of post-receptor ligation signaling. There are three major groups of MAP kinases in mammalian cells: the extracellular signal-regulated protein kinases (ERK 1 and ERK 2), the p38 MAP kinase and the c-Jun NH_2_-terminal kinases (JNK). ERK 1 and ERK 2 are 84% identical and share many functions. For this reason, they will be referred to here by the traditional designation, ERK1/2. Different cellular stimuli activate ERK1/2, which after stimulation, phosphorylates several key regulator proteins, including additional kinases, cytoskeletal proteins, nuclear receptors and several transcription factors involved in the transcription of cytokine genes. ERK1/2 modulates cell cycle progression, proliferation, cytokine production, transcription, differentiation, senescence and cell adhesion [19,20]. Little is known about the role of these signaling proteins during *S. mansoni* antigen activation.

The two-signal theory suggests that T-cell activation requires both antigen recognition via the TCR-CD3 complex and additional co-stimulatory signals derived from CD28 and other receptors. CD28 ligation can mediate increases in 3’phosphorylated inositol phospholipids by direct recruitment of the PI-3 kinase [21,22]. Perhaps the best studied of the PI-3 kinase targets are the Akt (or PKB) serine/threonine kinases. The downstream actions of Akt include the phosphorylation of proteins involved in the apoptotic cascade and regulation of the expression of apoptotic proteins [23]. Little is known about the involvement of MAPK and Akt during the *in vitro* PBMC immune response of individuals infected by *S. mansoni*. The objective of this study was to examine the effects of SEA and SWAP on T-cell activation, proliferation, cytokine production, and ERK1/2 and Akt phosphorylation in the PBMCs of infected and egg-negative individuals living in an *S. mansoni* endemic area in Brazil.

## Results

### Lymphocyte phenotyping

**Figure 1:**
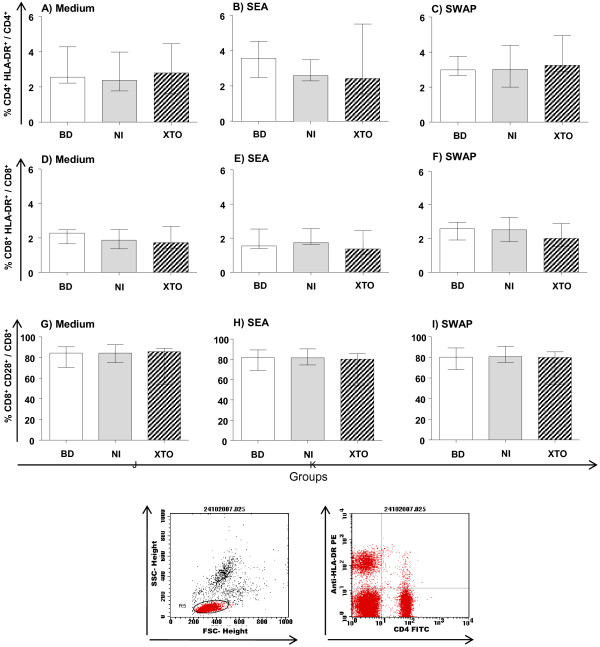
**Analysis of activated T-cell subpopulations after culture. **Flow cytometry was performed to assess the expression of the HLA-DR^+ ^activation marker on CD4^+^, CD8^+ ^and CD28^+ ^cells in peripheral blood. Lymphocyte phenotyping was performed to assess the activation of CD4^+ ^and CD8^+ ^cells after 120 hours of culture. PBMCs were stimulated with soluble egg antigen (SEA) and soluble worm antigen preparation (SWAP). Specific gating strategies to select the T cells subsets are represented by the dot plots (**J** and **K**). The results are expressed as the median (interquartile range) for each group BD: n = 11, NI: n = 13 and XTO: n = 10. The egg counts in the XTO group ranged from 12 to 96 eggs/g of stool (mean = 54 eggs/g). The Kruskal-Wallis and Dunn's test post-test were performed for statistical analysis.

The data showed that there is no difference in the expression of activation markers in the NI and XTO groups after culture compared to the BD group (Figure 1).

### Cell proliferation

**Figure 2:**
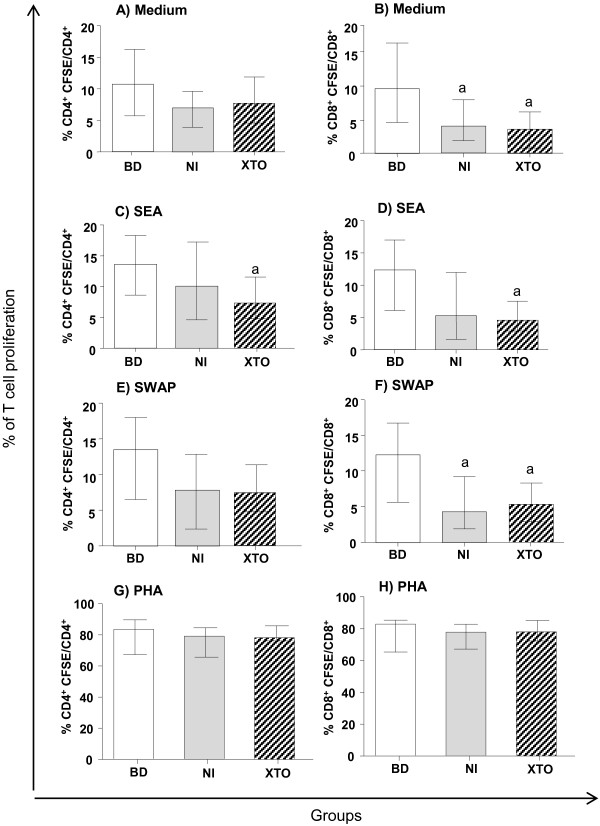
**Inter-group proliferation analysis of T-cell subpopulations.** Carboxyfluorescein diacetate succinimidyl ester (CFSE) flow cytometry was performed to assess the responsiveness of CD4^+ ^and CD8^+ ^cells after 120 hours of culture. PBMCs were stimulated with soluble egg antigen (SEA) and soluble worm antigen preparation (SWAP). The mitogen controls (phytohemagglutinin - PHA) of the CD4^+ ^and CD8^+ ^T cells are presented (**G **and **H**). The results are expressed as the median (interquartile range) for each group BD: n = 19, NI: n = 31 and XTO: n = 44. The egg counts in the XTO group ranged from 16 to 384 eggs/g of stool (mean = 200 eggs/g). The Kruskal-Wallis and Dunn's post-test were performed for statistical analysis. The letter "a" represents the difference (p <0.05) compared to BD group.

The CD8^+^ cell subpopulation of the NI group showed a lower proliferative response than the BD group in both the unstimulated cultures and the cultures stimulated with SWAP (Figure 2B and 2F). After SEA stimulation, CD4^+^ T cell proliferation was lower in the XTO group compared to the BD group (Figure 2C). CD4^+^ T cell proliferation was not different between the groups after either SWAP or medium stimulation (Figure 2A and 2E). CD8^+^ T cell proliferation was lower in the XTO group compared to the BD group after both SEA and SWAP stimulation and in the unstimulated cultures (Figure 2B, 2D and 2F). The inter-stimuli comparisons were not different (data not shown). The mitogen control (phytohemagglutinin - PHA) showed that both the CD4^+^ and CD8^+^ T cells were viable (Figure 2G and 2H).

### Cytokine response to *S. mansoni* antigens

#### Inter-stimuli comparisons

**Figure 3:**
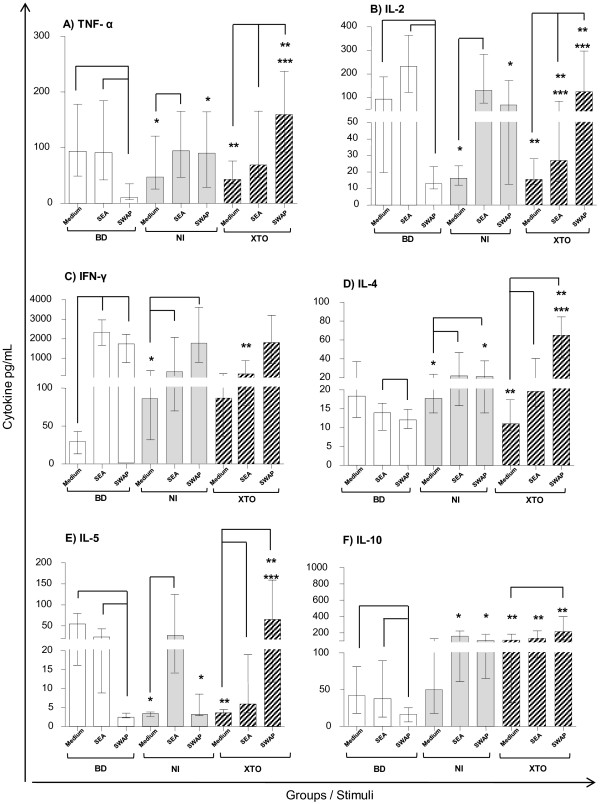
**Cytokine profile in the culture supernatants of PBMCs. **Levels of the cytokines (**A**) TNF-α, (**B**) IL-2, (**C**) IFN-γ, (**D**) IL-4, (**E**) IL-5 and (**F**) IL-10 were quantified by the CBA system and flow cytometry in the PBMCs of individuals from each group. BD: n = 19, NI: n = 21 and XTO: n = 27. The egg counts in the XTO group ranged from 17 to 68 eggs/g of stool (mean = 36 eggs/g). The bars represent the median and interquartile range. The Kruskal-Wallis and Dunn's statistical tests were performed for statistical analysis. The inter-stimuli differences (p <0.05) are represented by lines. The inter-groups differences (p<0.05) are represented by asterisks: BD x NI (*); BD x XTO (**); NI x XTO (***). The results are expressed in pg/mL.

All SEA and SWAP stimulated cultures were compared to the unstimulated cultures for the BD, NI and XTO groups. In the BD group, SWAP stimulation induced a decrease in TNF-α, IL-2, IL-4, IL-5 and IL-10 production compared to the unstimulated cultures. In some instances, there was a likewise comparison with the SEA-stimulated cultures (Figure 3). By contrast, there was an increase in IFN-γ after SWAP stimulation compared to the unstimulated cultures in the BD group.

The cytokine milieu showed that TNF-α production was higher in the NI group after SEA stimulation compared to the SWAP-stimulated and unstimulated cultures. The TNF-α level was higher in the XTO group after SEA and SWAP stimulation compared to the unstimulated cultures (Figure 3A). After SWAP stimulation, there was an increase in TNF-α secretion compared to SEA stimulation in the XTO group (Figure 3A).

In the NI group, SEA stimulation induced an increase in IL-2 compared to the unstimulated cultures. High levels of IL-2 were produced by SWAP stimulation compared to the SEA-stimulated and unstimulated cultures in the XTO group (Figure 3B). The IFN-γ levels were higher in the NI and XTO groups after SWAP stimulation than in the unstimulated cultures (Figure 3C).

IL-4 secretion was higher in the NI and XTO groups after both SEA and SWAP stimulation compared to the unstimulated cultures (Figure 3D). IL-5 production was higher in the NI group after SEA stimulation compared to the unstimulated cultures. The IL-5 level was higher in the XTO group after SEA and SWAP stimulation compared to the unstimulated cultures (Figure 3E).

High levels of IL-10 were observed in the XTO group after SWAP stimulation compared to the unstimulated cultures (Figure 3F).

#### Inter-group comparisons

Inter-group comparisons of cytokine production were made, and the results are shown in Figure 3. The significant differences are represented by asterisks.

TNF-α and IL-2 secretion in the unstimulated cultures was lower in the NI and XTO groups compared to the BD group. By contrast, after SWAP stimulation, an opposite profile was observed. In this case, there was an increase of TNF-α and IL-2 in the NI and XTO groups compared to the BD group (Figure 3A and 3B).

In the unstimulated PBMC cultures, IFN-γ secretion was higher in the NI group than the BD group. In the XTO group, IFN-γ secretion was lower compared to the BD group after SEA stimulation (Figure 3C).

IL-4 and IL-5 secretion were higher in the NI and XTO groups after SWAP stimulation compared to the BD group (Figure 3D and 3E). IL-10 secretion in the XTO group was higher than the BD group for both the SEA- and SWAP-stimulated or unstimulated cultures. In the NI group, IL-10 secretion was higher in the SEA- and SWAP-stimulated cultures than in the BD group (Figure 3F).

### Effects of SEA and SWAP on ERK1/2 and Akt phosphorylation in PBMCs

**Figure 4:**
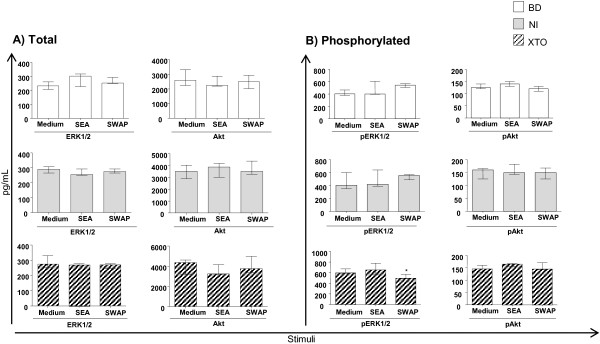
**Levels of ERK, pERK1/2, total Akt and pAkt after SEA or SWAP stimulation.** Inter-stimuli comparisons for pERK1/2 and pAkt were evaluated by phospho-ELISA of the BD group: n = 4, NI: n = 5 and XTO: n = 5. The egg counts in the XTO group ranged from 20 to 96 eggs/g of stool (mean = 36 eggs/g). The bars represent the median and interquartile range. The Kruskal-Wallis and Dunn's post- were performed for statistical analysis. The differences (p <0.05) are represented by an asterisk and are compared to the blood donor group (*). The results are expressed as pg/mL.

The inter-stimuli comparisons for total ERK1/2 and Akt levels were not different for any of the stimuli (Figure 4).

The levels of Akt were higher in the XTO group, in both the unstimulated cells and after SWAP stimulation, compared to the BD group. In the NI group, the Akt levels were increased after SEA stimulation (data not shown).

Inter-stimuli comparisons for pERK1/2 and pAkt showed that only the SWAP-stimulated PBMCs in the XTO group had reduced levels of ERK1/2 phosphorylation compared to the SEA –stimulated PBMCs (Figure 4B). No significant differences were observed for all the other comparisons.

## Discussion

### Lymphocyte phenotyping

In a previous study [24], an increase in HLA-DR expression was observed on CD8^+^ T cells without *in vitro* stimulation. However, when we evaluated the expression of activation markers in the NI and XTO groups after SEA and SWAP stimulation, (Figure 1) there was no increase of any marker compared to the BD group. This result suggests that there may be a relationship between activation and proliferation. This finding would explain the decreased lymphocyte proliferation after antigenic stimulation in individuals with a chronic infection [3]. The non-activation of PBMCs in the XTO and NI groups may be a result of constant stimulation by *S. mansoni* antigens that are present in endemic area, resulting in anergy.

### Cell proliferation

Cell proliferation is an early event in antigen-specific immune responses, and determining the percent proliferation is important for assessing the functional activity of immune cells and proliferation induced by *S. mansoni* antigens [6,11,25-27]. In patients during the chronic intestinal phase of schistosomiasis, cell proliferation is reduced in SEA-stimulated PBMCs compared to uninfected individuals [3]. Our previous findings [24] showed no difference between T cell proliferation in the XTO, NI and BD groups after both SEA and SWAP stimulation. However, the proliferation profile in this study is consistent with the findings of most other studies [3,4,27,28] that show lower CD4^+^ and CD8^+^ T cell proliferation in the XTO group than the BD group after SEA stimulation (Figure 2C and 2D). There was also decreased proliferation of CD8^+^ T cells after SWAP stimulation in the XTO group compared to the BD group (Figure 2F). The fact that the PBMCs from the NI and XTO patients were obtained from two distinct endemic areas may explain the differences between our studies. The population living in endemic areas is heterogeneous, and the immunological profile of each individual is a result of re-infection, delayed hypersensitivity and severity of the disease [5].

Moreover, the NI group showed reduced proliferation of CD8^+^ T cells after SWAP (Figure 2F) stimulation in the unstimulated cultures compared to the BD group (Figure 2B). The lower proliferation rate in the presence of SWAP in the NI group may be associated with increased synthesis of IL-4 and IL-5, which is caused by SWAP stimulation [10].

### Cytokine response to *S. mansoni* antigens

The mechanisms involved in the induction of Th1 and Th2 responses to schistosomiasis have not yet been fully elucidated, and the mechanisms that induce both responses are still under discussion. Some studies in murine models show that the eggs are responsible for a predominantly Th2 response. This finding is in contrast to the adult worms that appear to be weak Th2-inducers [29,30].

Our results showed that in the XTO group, SEA stimulation induced higher production of TNF-α, IL-2, IL-4 and IL-5 compared to the unstimulated cultures. SEA stimulation of the XTO group also decreased production of IFN-y compared to the SWAP-stimulated cultures (Figure 3). Compared to the unstimulated cultures, SWAP stimulation induced an increase of all the type 1 and type 2 cytokines analyzed in the supernatant of the PBMC cultures from subjects in the XTO group (Figure 3). A comparison between the SEA and SWAP-stimulated groups shows an increase in IFN-γ, which is consistent with the literature [12], and an increase in TNF-α, IL-2 and IL-5 levels. We suggest that in the XTO group either SWAP or SEA stimulation elicited a mixed immune response. These findings are in agreement with data previously published by our group showing that most of the chronic patients displayed a mixed type (type 1/type 2) immune profile [11,16,31].

Identification and characterization of *S. mansoni* antigens that can provide protective immunity is crucial for understanding the complex immunoregulatory events that modulate the immune response in schistosomiasis. In the chronic phase of schistosomiasis, TNF-α production was described as an important stimulator of nitric oxide production in response to the PIII-fraction of the soluble antigen of *S. mansoni* adult worms. NO induces a modulatory effect upon *in vitro* and *in vivo* granuloma formation. This finding suggests a protective effect related to immune response modulation [32,33]. Our data are consistent with the literature showing increased production of TNF-α after stimulation with SWAP in the supernatant of cultures from the XTO group (Figure 3A and 3F).

For the inter-group comparisons, the XTO group showed more IL-4, IL-5 and IL-10 secretion after SEA and SWAP stimulation compared to the BD group (Figure 3). These data are consistent with those previously published in which these cytokines may be related to the modulation of proliferation to *Schistosoma* antigens [10,11,34]. However, in the NI group, there was an increase in IL-2 and IFN-γ secretion compared to the BD group after SEA stimulation (Figure 3). Indeed, high levels of IFN-γ have been associated with resistance to schistosomiasis in individuals known as “endemic normal” [35-37]. High IFN-γ levels are also associated with resistance to infection after treatment [31]. These data may explain the increase in IFN-γ observed in the NI and BD groups. It has previously been shown that PBMC from blood donors produce high levels of IFN-γ concomitant with low levels of IL-10 after in vitro SEA stimulation, similar to what is observed during the acute phase of infection. Only after the second recall event, the immune response was polarized toward a strong Th2-like response [38].

We observed that the NI and XTO cells secreted significantly greater amounts of IL-10 compared to the BD group, in the unstimulated cultures and the SEA- and SWAP-stimulated cultures (Figure 3F). This may be an attempt to control the immune system through the production of other inflammatory cytokines. In fact, IL-10 is involved in the regulation of the human immune response during *S. mansoni* infection and has been associated with morbidity control [10,11,14,16,39-41]. In addition, the literature indicates that high IL-10 production is associated with the cellular response of patients during the asymptomatic chronic phase, whereas IL-10 production reduces the cellular response of patients during the hepatosplenic and acute phase [10]. More recently, it was demonstrated that in the presence of SEA, PBMCs from patients suffering from a chronic infection produce high amounts of IL-10 and secrete significantly lower levels of IFN-γ than uninfected individuals [12,28].

When we compared the synthesis of IL-10 in the NI and XTO groups, we observed an increase in IL-10 production independent of the stimuli, and the results were similar to those reported in the literature [42]. IL-10 can inhibit antigen presentation by dendritic cells. The mechanism of this response appears to be through blockade of certain cytokines, such as IFN-γ, IL-2 and TNF-α, modulation of the co-stimulatory molecules CD80, CD86 and MHC II and secretion of chemokines. In addition, the presence of IL-10 has a suppressive effect and limits the magnitude of activation [43].

Cytokines play an important role in cellular proliferation. In the comparisons between the different study groups, the XTO group showed less proliferation of both CD4^+^ and CD8^+^ T cells after SEA stimulation compared to the BD group. This may be associated with increased IL-10 production and reduced IFN-γ and IL-2 production in the XTO group. After SWAP stimulation, cell proliferation was lower for the CD8^+^ T cells, despite an increase in IL-2 and TNF-α and an increase in the secretion of IL-4, IL-5 and IL-10 compared to the database. Because the results of the proliferation and cytokine assays did not have any statistical correlation, further functional studies are needed to clarify the reduction in proliferation*.* Our data suggest that even though priming and culture conditions can skew a T-cell population toward the increased expression of some cytokines and the decreased expression of other cytokines, the expression of each cytokine can be regulated independently.

### Effects of SEA and SWAP on ERK1/2 and Akt phosphorylation

Few studies have sought to elucidate the molecular mechanisms involved in regulating the immune response in individuals infected with *S. mansoni*. [44]. ERK1/2 is involved in the cytotoxic activity of CD8^+^ T cells [45,46]. During T cell activation, anergic clones fail to activate ERK1/2; however, there is also evidence that inhibition of ERK1/2 alone cannot act on this unresponsive state known as clonal anergy [47].

In this study, ERK1/2 phosphorylation status may be associated with phenotypic results when there was no PBMC activation *in vitro*. Stimulation and co-stimulation promotes T cell proliferation, cytokine production, cell survival, and cellular metabolism through the activation of signaling pathways that send information to the nucleus [17]. If there was no surface receptor activation after SEA and SWAP stimulation, activation of the ERK1/2 signaling pathway activation may have been compromised.

Almeida et al. (2001) [44] have shown that after SWAP stimulation, the phosphorylation levels of Lck and Shc were more pronounced in the SWAP- than in the SEA-stimulated PBMCs. These data suggest that SEA and SWAP induce proliferation of lymphocytes both selectively and separately. Our results show that there was decreased phosphorylated ERK1/2 in the cells from individuals in the XTO group after SWAP stimulation (Figure 4B). Compared to that study, we hypothesize that the decrease in phosphorylated ERK1/2 may have occurred because of the involvement of Lck and Shc.

Phosphorylation analyses were performed *in vitro* after 120 hours of culture. The culture time was tested pre-viously by others [3,7], who showed that 120 hours of culture is necessary to observe the effect of the antigens on PBMCs. However, it is known that the duration of ERK1/2 activation depends on the type of stimuli [48-50]. It has been demonstrated in fibroblasts that a correlation exists between the signal intensity and duration of mitogen activation of ERK1/2. Furthermore, no mitogenic factors induce transient (15 minutes) activation of ERK1/2 that do not also induce cell cycle progression, whereas mitogen stimulation induces long-term activation of ERK1/2 (approximately 6 hours) [51,52]. Therefore, the stimulation time, the number of patients per group in the phosphorylation assay and the fact that SEA and the SWAP are not mitogenic stimuli may have influenced the phosphorylation state of ERK1/2.

There are currently no studies that have investigated the involvement of Akt in the immunomodulation of granulomas or the immune response in schistosomiasis. The similarity of Akt phosphorylation in the BD, NI and XTO groups may have occurred because of the low *in vitro* activation of CD28 after 120 hours of culture (data not shown). Phosphorylation of Akt may have followed the same pattern of CD28 activation.

All normal immune responses decrease with time, cau-sing the activation of regulatory mechanisms that are triggered by CTLA-4 expression [53]. Therefore, after 120 hours of culture, CD28 expression may have increased the expression of CTLA-4, which, in turn, may have generated a signal that does not increase Akt activation. This hypothesis should be confirmed by experiments that evaluate the expression of CTLA-4 under the same conditions *in vitro*.

We conclude that SEA and SWAP exert distinct effects on cell proliferation and cytokine production in the PBMCs of infected and egg-negative individuals living in the same endemic area. Infected individuals (XTO) present with reduced CD4^+^ T cell proliferation after SEA stimulation only. By contrast, CD8^+^ T cells do not seem to be significantly influenced by either the SEA or SWAP antigens because their proliferation was reduced in the unstimulated cultures in both the XTO and NI groups. The low CD4^+^ proliferation rate after SEA stimulation may be related to low secretion of IFN-γ and IL-2 and higher production of IL-10 in the XTO group compared to the BD group.

The influence of SWAP is clearly observed in the comparisons between the XTO and BD groups with regard to proliferation and cytokine production. In the BD group, there is low production of TNF, IL-2, IL-4, IL-5 and IL-10 as well as greater proliferation. The opposite is observed in the infected patients group. In this group, there is higher secretion of TNF, IL-2, IL-4, IL-5 and IL-10 and reduced proliferation compared to the blood donors group.

We also observed the influence of SWAP on ERK1/2 phosphorylation in the XTO group. It may be that in the first few hours of culture, ERK1/2, Akt or both may have been phosphorylated, which would influence cell proliferation. We are interested in determining the long-term significance of T cell hyporesponsiveness, with a particular focus on the relationship between the expression of surface regulatory markers and molecular activation.

## Conclusions

In the XTO group, it was observed that after SEA stimulation, there was a hyporesponsiveness of CD4^+^ and CD8^+^ T cells compared to the BD group. A balance between Th1 and Th2 cytokines was observed in the NI and XTO groups after both SEA and SWAP stimulation. The inter-group comparisons show that there was no difference in both ERK1/2 and Akt phosphorylation compared to the BD group.

## Methods

### Study population

This study was conducted with voluntary individual participation from the São Pedro Jequitinhonha and Virgem das Graças areas (Municipality of Ponto dos Volantes), located in a semi-arid, poor region of outmigration. Both are poor, rural areas that are hyperendemic for schistosomiasis. These areas are located in the Jequitinhonha Valley in northern Minas Gerais, Brazil. Virgem das Graças has the lowest Human Development Index (HDI) of 0.595 (United Nations Program for Development / UNDP, 2007) and a schistosomiasis prevalence of twenty-six percent. In São Pedro Jequitinhonha, the schistosomiasis prevalence was forty-seven percent. Two-hundred eighty people who lived in the urban São Pedro Jequitinhonha area participated in the parasitological survey. Eighty-seven of these individuals were selected for the tests performed in this study. In Virgem das Graças, five-hundred seventy people participated in survey, and twenty-four were selected and agreed to participate in this study.

Stool samples were collected for three consecutive days from each individual and were examined on duplicate slides to estimate the intensity of infection, as determined by the Kato–Katz fecal thick-smear technique [54]. The study population was classified as follows: infected patients presenting with *S. mansoni* eggs in their stool (XTO); egg-negative individuals (NI) living in the same endemic area as the XTO individuals and non-infected individuals recruited from blood donor volunteers (BD). The last group consisted of individuals who were born in and live in the capital of Minas Gerais (urban area) and who reported not having schistosomiasis. They ranged in age from eighteen to fifty years. These individuals were volunteers and their feces were not analyzed for the presence of *S. mansoni* and other parasites. The XTO group included patients ranging in age from fifteen to fifty years, and the NI group consisted of individuals ranging in age from fifteen to forty six years.

This study was reviewed and approved by the Centro de Pesquisas René Rachou, Fiocruz, the Federal University of Minas Gerais Ethics Committees (number ETIC001/09) and the Brazilian National Committee for Ethics in Research (CONEP). Written informed consent was obtained from all participants prior to the commencement of the study.

### Preparation of antigens

Soluble egg antigen from *S. mansoni* was prepared accor-ding to previously described methods [3,6]. Briefly, the eggs were collected from the livers of a laboratory population of out-bred Swiss mice infected with the LE strain of *S. mansoni*. The eggs were resuspended in 1.7% saline and subjected to homogenization in a tissue grinder for 30 seconds. This process was repeated three times each for 60 seconds. The resulting homogenate was centrifuged for 1 hour at 50,000 g, and the supernatant was collected and dialyzed against cold phosphate-buffered saline (PBS 0.15 M, pH 7.4). The protein concentration was determined by bicinchoninic acid assay (23227 Thermo Fisher Scientific Inc., Pierce Protein Research Products, Rockford, IL, USA). The optimal concentration of SEA for *in vitro* proliferation of lymphocytes has been standardized and reported [55]. Adult worms were collected by portal perfusion of out-bred Swiss mice 52 days after infection with 100 cercariae of the *S. mansoni* LE strain, and stored at −70°C. SWAP was prepared as previously described [3,6]. Briefly, 200 mg of adult worms were homogenized in a tissue grinder and centrifuged for 1 hour at 50,000 g. The supernatant was collected, dialyzed with cold PBS (0.15 M, pH 7.4), and the protein content was determined by the above method.

### Cell preparation and culture

Peripheral blood mononuclear cells were isolated from heparinized blood by density gradient centrifugation on Histopaque-1077 (Sigma-Aldrich, St. Louis, MO, USA) as previously described [3,6]. The cells were resuspended in RPMI-1640 (Gibco, Paisley, UK) medium at a final concentration of 1 × 10^7^ cell/mL. The cell culture experiments were performed in triplicate in 96-well microtiter plates for the proliferation assay. PBMCs (0.25 × 10^6^) were added to each well in complete RPMI-1640 containing 5% heat-inactivated normal human AB serum, antibiotic/antimycotic solution (100 U/mL penicillin, 100 μg/mL streptomycin, 0.25 μg/mL amphotericin-B; Sigma-Aldrich, St. Louis, MO, USA) and 2 mM L-glutamine (Winlab, Market Harborough, UK). The cells were incubated for 120 hours in the presence of 25 μg/ml of SEA, SWAP or medium alone (unstimulated). For phenotyping, cytokine and ELISA assays, 10^6^ PBMCs were added to each well in 24-well microtiter plates.

### Lymphocyte phenotyping

The cells were incubated for 120 hours in the presence of 25 μg/mL SEA, SWAP or medium alone (unstimulated). Then, cells were detached and mouse anti-human monoclonal antibodies (mAbs) conjugated with fluorescein isothiocyanate (FITC), phycoerythrin (PE), or tri-color (TC) specific for cell-surface markers were used simultaneously in two-color flow cytometric assays. The first color reagents consisted of anti-human FITC-conjugated anti-CD4 mAbs (L200) or anti-human TC-conjugated anti-CD8 mAbs (3B5) with mouse IgG1 as the isotype control (MOPC-21). The second color reagents included anti-human PE-conjugated anti-HLA-DR (G46-6) and anti-CD28 (CD28.2) with mouse IgG2 as the isotype control (G3–245). All antibodies were purchased from BD Biosciences (San Jose, CA, USA), with the exception of TC-conjugated anti-CD8 mAbs, which were obtained from Caltag Laboratories (Burlingame, CA, USA). A sample of 1 × 10^6^ PBMC was washed in PBS, stained with the appropriate antibodies, rewashed and fixed for 10 minutes at room temperature with a fluorescence-activated cell sorter (FACS) fixing solution. A minimum of 20,000 cells per sample was analyzed in a FACScan flow cytometer (BD FACScan_ Flowcytometer; BD Biosciences). Selective analysis of lymphocytes was performed by placing an electronic gate on the forward angle light scatter (FSC) X side angle light scatter (SSC) dot plot for small blood lymphocytes. A selective window within the small lymphocyte gate was established on a specific fluorescent population to further focus on the activation states of either the CD4^+^ or CD8^+^ subpopulations. This assay was performed with samples from eleven blood donors (BD: n = 11), thirteen egg-negative (NI: n = 13) and ten infected individuals (XTO: n = 10). The egg counts in the XTO group ranged from 12 to 96 eggs/g of stool (mean = 54 eggs/g).

### Cell proliferation assay

CellTrace™ CFSE stock solution (code C34554, Molecular Probes- Invitrogen, Eugene, OR, USA) was prepared in dimethyl sulfoxide according to manufacturer's instructions. This solution was diluted 1/1000 in PBS. A sample of 5 × 10^6^ PBMC was added to 500 μL of CFSE solution and incubated at room temperature for 10 minutes in the dark. The staining was then quenched by the addition of 1 mL 10% RPMI-1640/FSC for 5 minuntes on ice. The cells were washed twice in DMEM medium and centrifuged at 4°C at 400 × g for 10 minutes. Next, the PBMCs were resuspended in the above medium and cultured in a 96-well microtiter plate. This assay was performed with samples from nineteen blood donors (BD: n = 19), thirty-one egg-negative (NI: n = 31) and forty-four infected individuals (XTO: n = 44). The egg counts in the XTO group ranged from 16 to 384 eggs/g of stool (mean = 200 eggs/g).

### Cytokine measurement assay

At the end of 120 hours, the culture supernatant was collected and immediately frozen at −70°C for subsequent determination of cytokine production. The assay was performed by a flow cytometry application that allows us to quantify multiple cytokines simultaneously. IL-2, IL-4, IL-5, IL-10, IFN-γ and TNF-α were measured in the supernatant using the Cytometric Bead Array method (Human Th1/Th2 Cytokine CBA kit, BD, Pharmingen, USA) as recommended by the manufacturer and described previously. Briefly, a mixture of beads specific for human cytokines each with distinct fluorescence intensities (in the FL-3 channel) was coated with capture antibodies specific for each cytokine. A second, fluorescently labeled PE-anti-cytokine antibody was added, and the levels of the individual cytokines were indicated by their Mean Fluorescence Intensity (MFI). The results were expressed as pg/mL and were calculated from standard curves for each cytokine. Data were acquired on a FACScalibur flow cytometer (Becton Dickinson), and the analyses were performed using the BD CBA software (Becton Dickinson). This assay was performed with samples from nineteen blood donors (BD: n = 19), twenty-one egg-negative individuals (NI: n = 21) and twenty-seven infected individuals (XTO: n = 27). The egg counts in the XTO group ranged from 17 to 68 eggs/g of stool (mean = 36 eggs/g).

### Phosphorylation assay

At the end of 120 hours, the cultured cells were collected in cold PBS and centrifuged at 14,000 rpm. The supernatant was collected and the cells were resuspended in 1 mM Na_3_VO_4_ (Sigma Aldrich) solution containing Complete Protease Inhibitor (Roche) and frozen at −70°C. Phosphorylation of Akt and ERK1/2 was assessed in the cell extracts by ELISA assay (Invitrogen). This method quantifies the amount of Akt that is phosphorylated at serine residue 473. It also quantifies the dual-phosphorylated ERK1/2 that is phosphorylated at the threonine 185 and tyrosine 187 residues. This assay was performed with samples from four blood donors (BD group: n = 4), five egg-negative individuals (NI: n = 5) and five infected individuals (XTO: n = 5). The egg counts in the XTO group ranged from 20 to 96 eggs/g of stool (mean = 36 eggs/g).

### Statistical analysis

Statistical analyses were performed using GraphPad Prism version 4.00 for Windows (GraphPad Software, San Diego, CA, USA). Comparisons between the three groups with respect to the medians of the data exhibiting non-parametric distributions were performed with the Kruskal–Wallis test. Dunn’s post-test was used for multiple comparisons. The results are presented as the median (interquartile range) in the figures. The confidence intervals were set at the 95% level (p < 0.05).

## Abbreviations

SEA: Soluble egg antigen; SWAP: Soluble worm antigen preparation; PBMC: Peripheral blood mononuclear cells; NI: Egg-negative individuals; XTO: Infected individuals; BD: Blood donor healthy individuals.

## Competing interests

There are no financial competing interests.

## Authors’ contributions

ROP is the first author and has made substantive contributions, including the acquisition, analysis and interpretation of data, statistical analyses and drafting of the manuscript. IRC, ATC, and RCO participated in the design of the study. RCGF and LMP contributed to the immunoassays, cell culture and cytokine assays. AG coordinated all the steps in obtaining the samples from the endemic area, including collection of stool samples and blood samples and explanation of the study to all participants with informed consent. MVA gave final approval of the manuscript and coordinated the signaling assays. JRCM was responsible for coordination of the study and helped to draft the manuscript. All authors read and approved the final manuscript.

## Pre-publication history

The pre-publication history for this paper can be accessed here:

http://www.biomedcentral.com/1471-2334/12/380/prepub
